# RNA m^6^A Modification in Immunocytes and DNA Repair: The Biological Functions and Prospects in Clinical Application

**DOI:** 10.3389/fcell.2021.794754

**Published:** 2021-12-20

**Authors:** Mingjie Zhou, Wei Liu, Jieyan Zhang, Nan Sun

**Affiliations:** ^1^ Department of Blood Transfusion, The Fourth Hospital of Hebei Medical University, Shijiazhuang, China; ^2^ Department of Immunology, Hebei Medical University, Shijiazhuang, China; ^3^ Department of Hand Surgery, Huashan Hospital, Fudan University, Shanghai, China; ^4^ Department of Orthopaedics, Wuxi Branch of Zhongda Hospital Southeast University, Wuxi, China

**Keywords:** m^6^A (*N* ^6^-methyladenosine), immunocyte, epigenetics, immunotherapy, DNA repair

## Abstract

As the most prevalent internal modification in mRNA, *N* ^6^-methyladenosine (m^6^A) plays broad biological functions *via* fine-tuning gene expression at the post-transcription level. Such modifications are deposited by methyltransferases (i.e., m^6^A Writers), removed by demethylases (i.e., m^6^A Erasers), and recognized by m^6^A binding proteins (i.e., m^6^A Readers). The m^6^A decorations regulate the stability, splicing, translocation, and translation efficiency of mRNAs, and exert crucial effects on proliferation, differentiation, and immunologic functions of immunocytes, such as T lymphocyte, B lymphocyte, dendritic cell (DC), and macrophage. Recent studies have revealed the association of dysregulated m^6^A modification machinery with various types of diseases, including AIDS, cancer, autoimmune disease, and atherosclerosis. Given the crucial roles of m^6^A modification in activating immunocytes and promoting DNA repair in cells under physiological or pathological states, targeting dysregulated m^6^A machinery holds therapeutic potential in clinical application. Here, we summarize the biological functions of m^6^A machinery in immunocytes and the potential clinical applications *via* targeting m^6^A machinery.

## Introduction

While RNA modification was first identified in 1970s, it becomes a research focus in recent years. It broadly exists in different species, including fungi (Bodi et al., 2015), plants (Yue et al., 2019), and animals (Yoon et al., 2017; Xia et al., 2018). During the past decades, researchers have found that RNA methylation is a widespread modification in coding sequence and non-coding sequence (Huang et al., 2020), most of which are located at the amine group outside ring, special nitrogen and carbon positions of purine and pyrimidine, and the oxygen atom of the 2′-OH moiety (Liu and Jia, 2014). If classified by the modified position, RNA methylation mainly consists of N^6^-methyladenosine (m^6^A), 5-methylcytosine (m^5^C), N^7^-methylguanosine (m^7^G), etc., among which m^7^G cap at the 5′end of RNA sequence has been rigorously studied for decades (Devarkar et al., 2016; Pandolfini et al., 2019). However, m^6^A modification, representing the most abundant modification, needs further study.

As reported, m^6^A modifications are localized in the conversed DRACH motifs (D = G/A/U, R = G/A, H = A/U/C). The distribution of m^6^A is usually in the coding and 3′ untranslated regions, especially enriched in the upstream of stop codon in mRNA (Roundtree et al., 2017). Recent researches find that m^6^A modification is also an important biological mark of endogenous circular RNA (circRNA) (Chen et al., 2019). Moreover, m^6^A modification in lncRNA can regulate the efficiency of glycolysis (Liu J et al., 2019) or promote oncogenesis (Chen et al., 2020). Since m^6^A modification is dynamic and reversible, the biological function and molecular mechanism of m^6^A modification have become a research hotspot in many medical fields.

## Writers, Erasers, and Readers

The most momentous breakthrough in this field is the discovery of the m^6^A machinery involved in m^6^A modification, including “Writers,” “Erasers” and “Readers,” performing the function of methyltransferase, demethylase, and recognizing the m^6^A structure, respectively. They dynamically regulate the homeostasis of m^6^A and its functions in cells.

### Writers

With the function of forming m^6^A structure, “Writers” protein is a 1 MDa complex composed of multiple subunits, containing Methyltransferase like-3 (METTL3) (Liu et al., 2014), Methyltransferase like-14 (METTL14) (Weng et al., 2018), Wilm’s Tumor 1-associating protein (WTAP) (Ping et al., 2014; Sorci et al., 2018), etc. METTL3 is responsible for catalyzing the transfer of methyl group with the support of S-adenosyl-methionine (SAM) in many types of RNA including mRNA and miRNA, while METTL14 is a catalytic cofactor capable of recognizing and binding the target mRNA. WTAP is in charge of recruiting the targeting RNA and locating the METTL3/METTL14/WTAP complex into the nuclear speckles, which is relevant to the prognosis and cisplatin resistance (Ma et al., 2021) of cancer and the infiltration of T lymphocyte within tumors (Li H et al., 2020). New subunits, termed RBM15/RBM15B (Knuckles et al., 2018), KIAA1429 (Lan et al., 2019), ZFP217 (Song et al., 2019), and ZC3H3 (Silla et al., 2020), have been identified, and their functions involve in the recruitment, m^6^A modification of mRNA or lncRNA, and regulation of the m^6^A catalytic efficiency. Different types of “Writers” may interact with each other, as a result of which may influence the progression of some diseases such as colorectal cancer (Chen H et al., 2021).

### Erasers

The m^6^A structure can be erased by the “Erasers” protein. Fat mass and obesity-associated protein (FTO) (Jia et al., 2011) was supposed to be the first demethylase discovered, whose existence confirmed the reversibility of m^6^A modification. FTO and the second identified “Erasers” called AlkB Homolog 5 (ALKBH5) (Zheng et al., 2013) jointly counter the m^6^A modification of “Writers,” thus maintaining the homeostasis of m^6^A level in cells, whereas the distribution of the two proteins are tissue-specific. The amino acid sequence HXDXnH and RXXXXXR (X = any amino acid) with demethylase activity are contained in their mutual AlkB domain. Both of them remove the m^6^A methylation from mRNA with the Fe (II)/*α*-ketoglutarate-dependent dioxygenase (Fedeles et al., 2015). The demonstration of “Writers” and “Erasers” initiates a new branch, namely, m^6^A research, in the field of epigenetics. Recent studies on ALKBH5 gradually elucidate its multiple functions in disease progressing and therapeutic efficacy, including CD4^+^ T cell pathogenicity in autoimmunity (Zhou et al., 2021), anti PD-1 response in tumor treatment (Li N et al., 2020), glucocorticoid resistance in T-cell acute lymphoblastic leukemia cell treatment (Gong et al., 2021), etc.

### Readers

The level of m^6^A in cells is dynamically modulated by “Writers” and “Erasers,” while “Readers” can recognize the m^6^A structure and regulate the subsequent cell processes such as translation and stability of mRNA. The YTH domain-containing family is the first confirmed component of “Readers,” characterized by the YTH domain at C terminus. YTHDF1∼3 and YTHDC1∼2 (Kasowitz et al., 2018; Zhou et al., 2020) are identified as m^6^A binding proteins, among which researches concerning YTHDF are more detailed. Generally speaking, the aforementioned m^6^A “Readers” proteins have the same function of binding the m^6^A-modified mRNA with the consensus YTH domain at C terminus, while YTHDF1 promote translation by binding the m^6^A at translation initiation site (Zhuang et al., 2019); YTHDF2, characterized by the P/Q/N-rich domain at N terminus, recruits the CCR4-NOT deadenylase complex and brings the target mRNA to cytoplasmic P bodies (Du et al., 2016), resulting in the destabilization of mRNA (Paris et al., 2019); YTHDF3 is also related to mRNA decay, but it is regarded to have a synergistic effect on YTHDF1 and YTHDF2 (Ni et al., 2019). In contrast to YTHDF, additional Readers such as insulin-like growth factor 2 mRNA-binding proteins (IGF2BPs) (Hanniford et al., 2020) can uniquely stabilize the target mRNA, while the eukaryotic initiation factor 3 (eIF3) (Meyer et al., 2015; Wolf et al., 2020) can promote cap-independent translation of mRNA with 5′-UTR m^6^A modified. Moreover, other m^6^A Readers like ELAVL1 (Zhang et al., 2017) are being studied recently.

## Roles of m^6^A Modification in Immunocytes

Immunocytes play a crucial role in a variety of bioprocesses, such as recognizing and presenting the pathogen and immune response, whose depletion or dysfunction is the important pathological basis of tumorigenesis, viral infection, and autoimmune diseases, etc. Previous researches focused on the function of m^6^A in cancer cells, including endometrial cancer (Liu et al., 2018), breast cancer (Cai et al., 2018), bladder cancer (Cheng et al., 2019), hepatocellular cancer (Zhao X et al., 2018), nasopharyngeal cancer (Zhang et al., 2018), glioblastoma stem cells (Cui et al., 2017), acute myeloid leukemia (Cui et al., 2017), etc. Nevertheless, recent m^6^A researches on T lymphocyte, B lymphocyte, DC, and macrophage broaden our cognition towards the human immune system, which are the latest achievements of m^6^A modification.

### T Lymphocyte

T lymphocyte is the executant of human adaptive immune system, which is related to antitumor immunity and autoimmune diseases. Furthermore, T lymphocyte is likely to have interaction with neural stem cells, thus inhibiting its proliferation and resulting in age-related brain disease (Dulken et al., 2019); the abnormal level of m5C, another form of RNA methylation, in CD^4+^ T lymphocytes may have a potential link with the pathogenesis of systemic lupus erythematosus (SLE) (Guo G et al., 2020). According to the differences in function and phenotype, T lymphocyte can be divided into subtypes including naïve T cell (Tn), cytotoxic T cell, regulatory T cell (Treg), helper T cell (Th), etc., which is mainly driven by the stimulation of inflammation factor such as interleukin (IL) and tumor necrosis factor (TNF). Recently, bile acid has been proved to be an accessional regulator of Th17 (Hang et al., 2019) and Treg differentiation. The newly discovered subtype termed exhausted T cell (Tex) along with its key transcription factor called thymocyte selection-associated high mobility group box (TOX) (Scott et al., 2019) is a breakthrough in antitumor study, although the connection between Tex and m^6^A remains to be explored.

**FIGURE 1:**
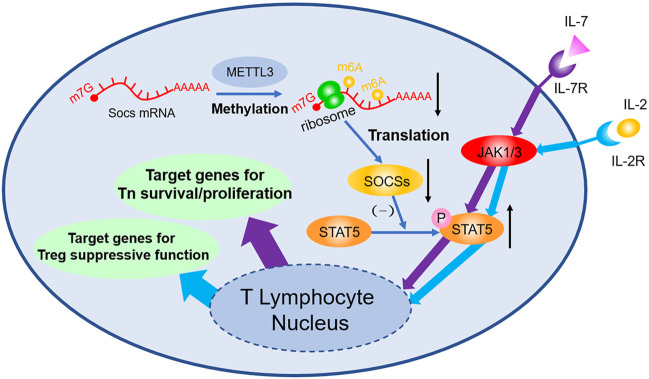
m^6^A decoration contributes to the regulation of Tn differentiation and Treg function both through JAK-STAT5 pathway. m^6^A modifications in *Soc1*, *Soc3*, and *Cish* mRNA accelerate their degradation, thus decreasing the expression of SOCS. As a consequence, through the JAK/STAT5 pathway, IL-7 induced differentiation of Tn and IL-2 induced suppressive function of Treg are both influenced afterwards.

Studies focusing on m^6^A in T lymphocyte mark the initiation of m^6^A study in adaptive immune field. Li H-B et al. (2017) found that 5 weeks after transplant of wild type Tn, Rag^−/−^ mice develop colitis due to the differentiation of Tn into effector T cell, whereas Rag^−/−^ mice with Mettl3^−/−^ transplanted exhibit no sign of similar symptoms and no T cell infiltration or inflammation inside spleen and colon can be observed. FACS shows the dysfunction of Mettl3^−/−^ Tn differentiation. Molecular biology studies indicate that *Soc1*, *Soc3*, and *Cish* mRNA are stabilized owing to the lack of m^6^A modification; afterwards, elevated SOCS protein inhibits the phosphorylation of STAT5, then the IL-7 mediated JAK-STAT5 pathway will be blocked, and thus the differentiation of Tn is suffocated. However, because Mettl3^−/−^ strengthen ERK and APK pathway simultaneously, no obvious increase in T cell apoptosis can be observed. Follow-up study (Tong et al., 2018) found that Tn homeostasis of Mettl3^f/f^; CD4-Cre mice can be destroyed and have colitis 3 months after being born, because Treg’s suppression of effector T cell is faulted. During this process, genetic depletion of *Mettl3* reduces the m^6^A modification of *Socs* mRNA, then stabilizes mRNA, and upregulates its expression. High level SOCS protein inhibits IL-2-STAT5 pathway, resulting in the dysfunction of Treg. Additionally, it is demonstrated that Treg can strengthen type-II DC’s ability of presenting antigen (Binnewies et al., 2019), enhance antitumor response, and improve prognosis of checkpoint blockade such as PD-1 block immunotherapy (shown in Figure 1).

Another study (Lu et al., 2020) unveils the function of *Mettl14* in T lymphocytes. In this research, CD4-Cre^+/Tg^ Mettl14^FL/FL^ conditional knockout mice have found to develop spontaneous colitis due to the increased level in Th1 cytokines, such as IFN-γ and TNF-α. Follow-up studies show that RORγt expression in *Mettl14* deficient Tregs is downregulated compared to the wild-type Tregs and the induction efficacy of *Mettl14* deficient Tn to iTreg is obviously impaired. As a consequence, both the reduction of iTregs, whose function has reported to be controlling the experimental colitis, and the dysfunctional Mettl14 deficient Tregs lead to the development of spontaneous colitis. With the function of METTL3 in T follicular helper cell differentiation being clarified recently (Yao et al., 2021), plenty of evidences have persuade us that m^6^A may play an irreplaceable role in all subtypes of T cells.

Furthermore, in acute myeloid leukemia (AML), FTO inhibition can lead to downregulation of leukocyte immunoglobulin-like receptor subfamily B member 4 (LILRB4), render AML cells vulnerable to activated T cells, and simultaneously overcome hypomethylating agent (HMA)-induced immune evasion. FTO’s function in anti-tumor immunity, especially its function in T cells, can be a promising therapeutic strategy in the field of m^6^A research (Su et al., 2020).

**FIGURE 2:**
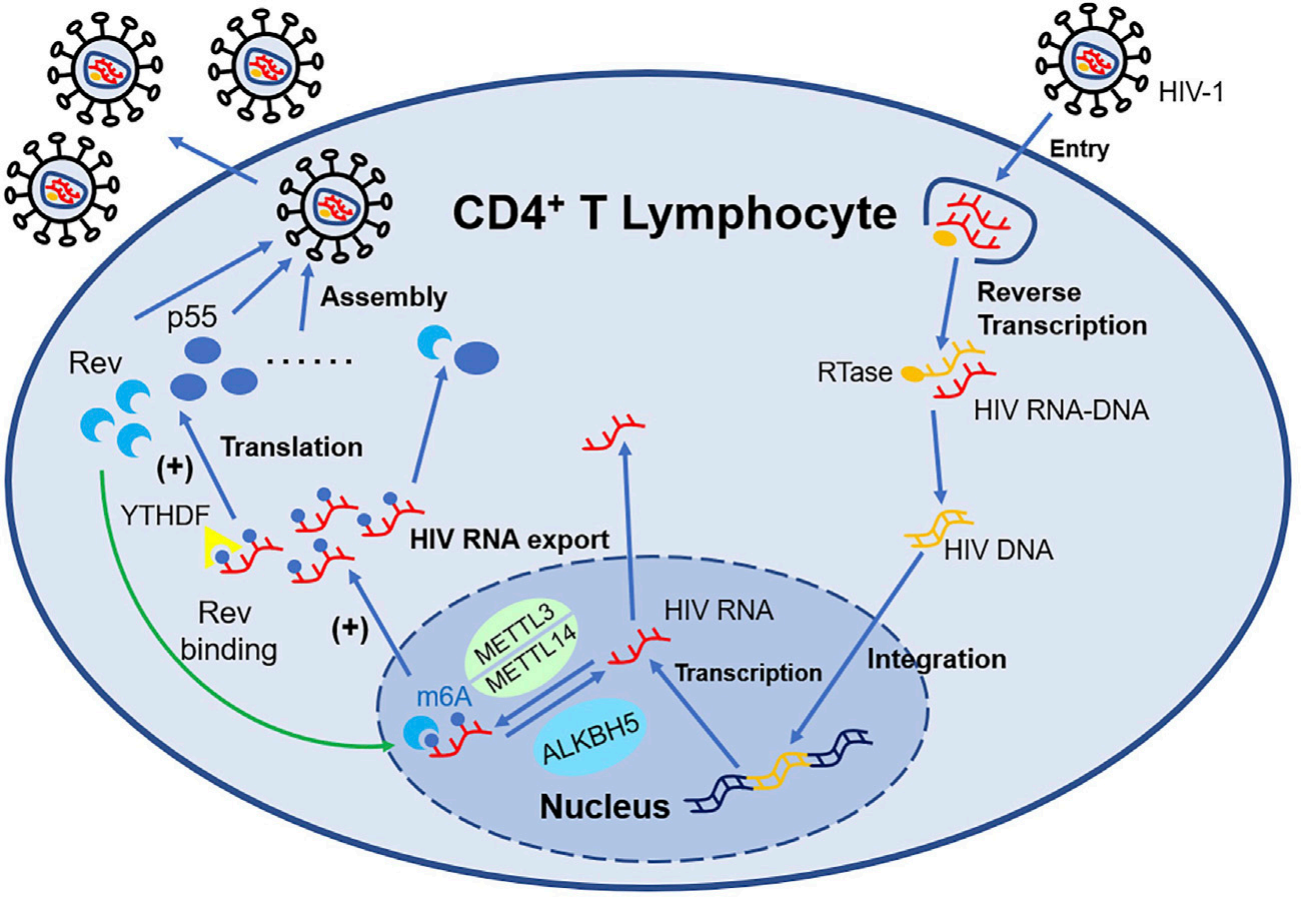
m^6^A-associated proteins in HIV-infected T lymphocytes regulate the expression of HIV mRNA. METTL3/METTL14 can install m^6^A decoration on HIV mRNA, while ALKBH5 can remove such modification. YTHDF1-3 recognize the m^6^A structure and influence the expression of HIV mRNA such as Rev and p55. With the support of Rev, HIV mRNA modified by m^6^A can export nucleus more easily, which promote the replication of HIV.

In addition, when CD4^+^ T cell is infected by the Human Immunodeficiency Virus (HIV), both HIV RNA and intrinsic RNA will be obviously increased (Lichinchi et al., 2016) independent of virus replication. Only gp120 will upregulate the level of m^6^A without influencing the expression of Writers and Erasers (Tirumuru and Wu, 2019). The m^6^A modification position of HIV RNA mainly distributes at 3′-UTR (Kennedy et al., 2016), which is directly involved in the regulation of viral mRNA nuclear export and protein synthesis. The m^6^A modification machinery, such as YTHDF (Tirumuru et al., 2016), METTL3/METTL14, and ALKBH5 (Lichinchi et al., 2016), act as a crucial regulator of this process. The m^6^A modification of HIV RNA will affect the expression of viral proteins including p55 (the product of gene *gag*) and Rev, consequently impacting the viral infectivity and replication, while suppressing the expression of IFN-I in monocytic cells and macrophages at the same time (Chen S et al., 2021). Therefore, antibodies neutralizing gp120 or CD4^+^ probably have the potential to counter HIV. Besides, Fu et al. (2019) for the first time applied HIV transgenic rats for m^6^A research and elucidated the role of m^6^A modification in mRNA in chronic HIV diseases, especially neurologic disorder (shown in Figure 2).

In conclusion, the effect of YTHDF2 on virus remains to be ascertained or can be bidirectional (Toro-Ascuy et al., 2016; Lu W et al., 2018); YTHDF3 weakens the viral infectivity and inhibits the viral replication. Uniquely, YTHDF3 can be incorporated into the virion and still keep its antiviral activation, but the HIV protease can cleave the virion containing YTHDF3. This mechanism prevents HIV from being killed thoroughly and provides a new thought for HIV treatment (Jurczyszak et al., 2020).

All the researches above reveal that m^6^A along with associated protein can alter the stability of mRNA and regulate nuclear export and translation of mRNA, thus influencing the bioprocess of T cell with different phenotype and promoting the progression of certain diseases.

### B Lymphocyte

B lymphocyte is involved in the humoral immunity by producing antibodies. Applying bioengineering technology to design special improbable immunogen can induce the synthesis of antibodies with high affinity, which have the potential to treat virus infection, such as HIV (Saunders et al., 2019). A recent clinical study suggests that m^6^A modification is closely related to the oncogenesis and progress of mantle cell lymphoma (Zhang W et al., 2019). Mantle cell lymphocyte is a kind of non-hodgkin B cell lymphoma characterized by aggressive phenotype and rapid rate of progression. After analysis of 123 samples of clinical patients, the hazard ratios of YTHDF3, METTL3, FTO, METTL14, ALKBH5, YTHDF2, and WTAP are below 1, while those of YTHDF1, KIAA1429, and ELAVL1 are above 1, among which the maximum is ELAVL while the minimum is YTHDF3, implying that ELAVL and YTHDF3 might be the most important regulators of mantle cell lymphoma. Moreover, “m^6^A index” is proposed to evaluate the prognosis of patients. Without much available biology research data, this statistical study directs a path for the following m^6^A research concerning B lymphocyte.

**FIGURE 3:**
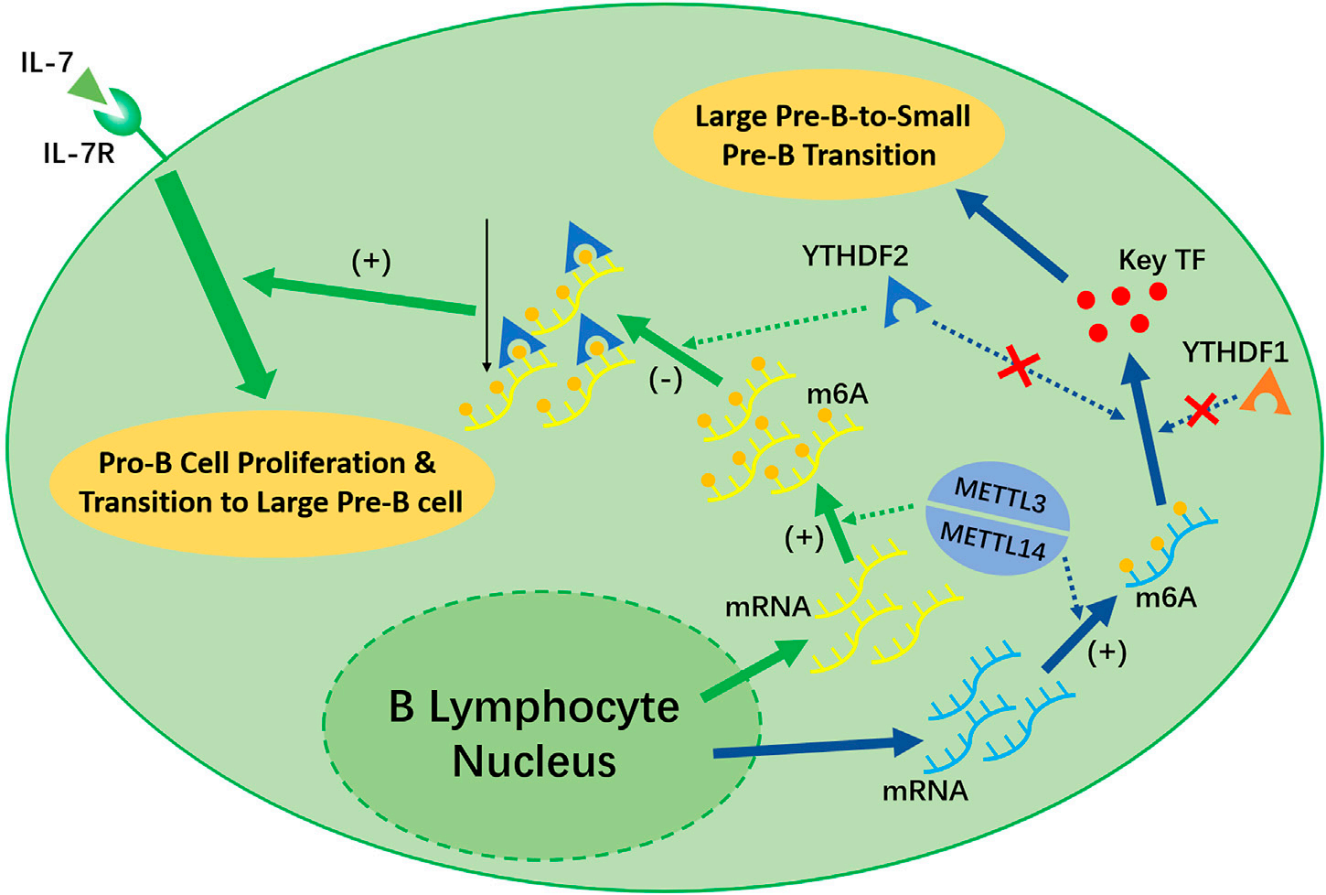
METTL3/METTL14 complex and YTHDF1 is necessary for the early development of B lymphocytes. METTL14 plays an unreplaceable role in the IL-7 induced Pro-B lymphocyte proliferation, Pro-B-to-Large-Pre-B transition, and Large-Pre-B-to-Small-Pre-B transition. YTHDF2 will recognize the m^6^A modification afterwards and decrease the transcripts as a result, which promote early B lymphocyte development. Notably, YTHDF2 only facilitate in the first two process. The Large-Pre-B-to Small-Pre-B Transition is independent of YTHDF1 or YTHDF2.

Recent studies have shown that the deletion of *Mettl14* can decrease the m^6^A level in developing B cells and inhibit some important processes, such as the IL-7-induced Pro-B cell proliferation and the transition to the Large Pre-B Stage, which depends on the function of YTHDF2 (Zheng et al., 2020) (shown in Figure 3). However, the Large-Pre-B-to-Small-Pre-B Transition depends on METTL14, but is independent of YTHDF1 or YTHDF2 (shown in Figure 3).

The pathogenesis of diffuse large B-cell lymphoma (DLBCL), which is the most common subtype of lymphoma derived from B lymphocytes, has been illustrated to have a link with upregulated METTL3 and the m^6^A level of the mRNA of pigment epithelium-derived factor (Cheng et al., 2020), while PIWI-interacting RNAs have been identified to function in this process recently (Han et al., 2021). The association between B lymphocytes and other m^6^A methylation-related proteins remains to be explored.

Besides, Kaposi’s sarcoma (KS) is evidently associated with infection of HIV and Kaposi’s sarcoma-associated herpesvirus (KSHV). KSHV shows strong lymphotropic and invades B cells in the circulation (Myoung and Ganem, 2011). During this process, m^6^A Reader protein YTHDF2 plays a positive role in KSHV replication (Hesser et al., 2018). However, inconsistent with its feature *in vivo*, KSHV exhibits weaken infectivity and proliferation in B cells lines *in vitro*, so the role of m^6^A and associated protein in the oncogenesis of KS remains to be explored.

### DC

As the bridge between the innate and adaptive immune, DCs function as antigen-presenting cells and can also produce VEGF-α for the recruitment of neutrophil to control cutaneous bacterial infections (Janela et al., 2019). Therefore, DCs play a core role in the eradication of pathogen and inducement of immune tolerance. It has been evidenced that the dysfunction of DC activation is involved in the progression of multiple inflammation, cancer, and autoimmune diseases. Tyrosine kinase AXL can induce the expression of PD-1. IFN-γ and IL-4 can respectively promote and inhibit the production of IL-12; thus, blockade of IL-4 receptor can strengthen antitumor response by expanding the infiltration of T lymphocyte at tumor position (Maier et al., 2020). Though the association between the suppression of DCs and extracellular m^6^A-modified RNA has been confirmed for decades (Karikó et al., 2005), studies concerning m^6^A in DCs are still at its infant stage.

**FIGURE 4:**
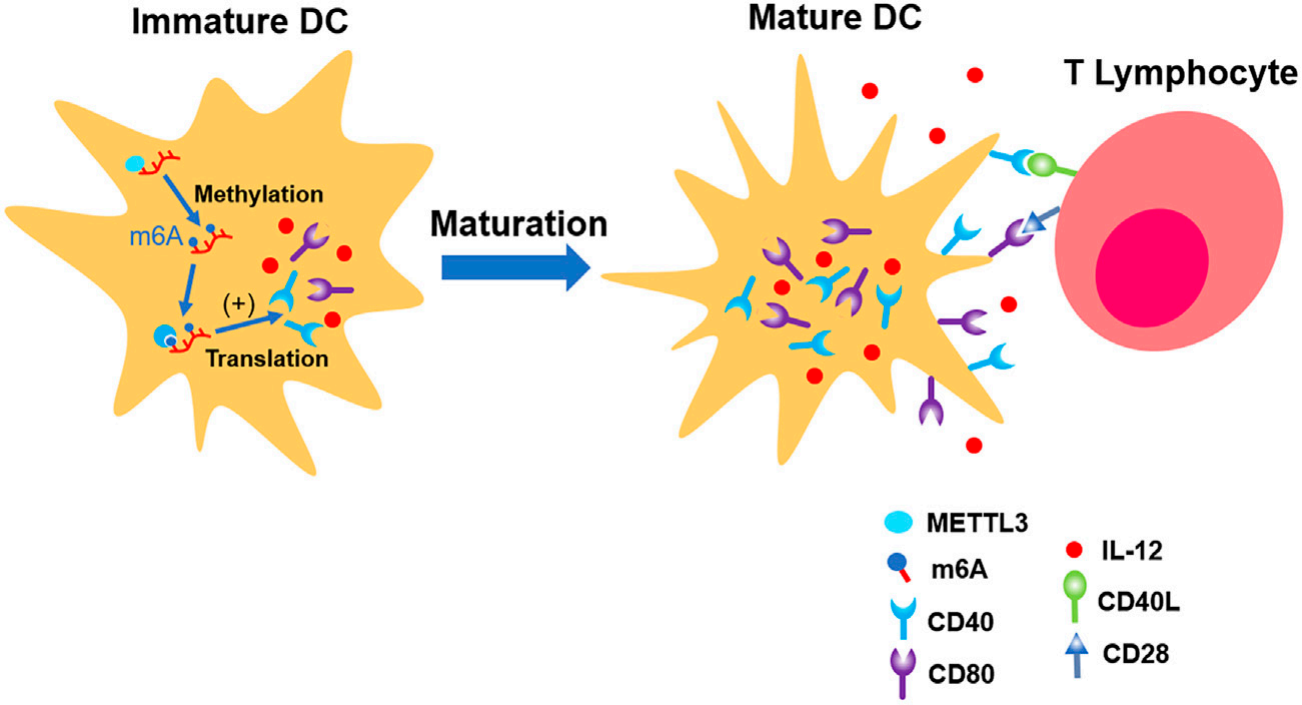
METTL3 up-regulates the expression of CD40, CD80, and IL-12, which promote the maturation of immature DC. In the end, its ability of presenting antigen and interaction with T lymphocytes are strengthened.

Wang H et al. (2019) found that total level of m^6^A in DCs is increasing parallel with its maturation. The distribution of m^6^A is mainly located in NLR, TNF, and NF-κB pathways, which are responsible for the induction of co-stimulatory factors and pro-inflammation factors which promote maturation of DCs. METTL3 was involved in this physiological process. Distinct from most of the previous laboratory findings, the fundamental mechanism is the upregulation of translation efficiency, but not the stability of mRNA (Wang H et al., 2019) (shown in Figure 4).

**FIGURE 5:**
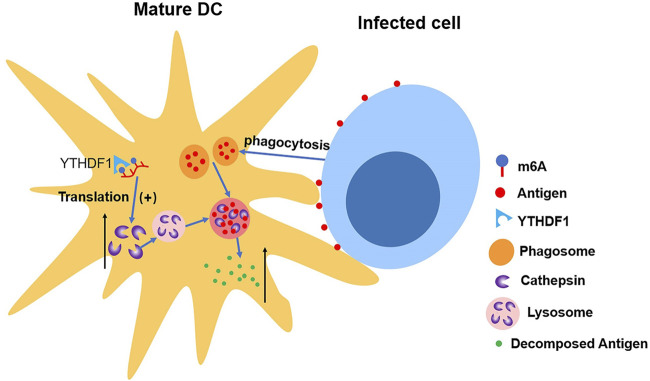
YTHDF1 increases the level of cathepsin in mature DCs, which accelerates the cleavage of antigens in phagosome, making DCs’ ability of presenting antigen impaired.

Han et al. (2019) discovered the connection between upregulated translation and YTHDF1. The depletion of YTHDF1 in DCs will limit the expression of lysosomal protease, which decelerates the degradation of antigen, thus improving DCs’ ability of presenting antigen and activating CD8^+^ T cell. This suggests a new mechanism of immune escape as well as an important reason for weak antitumor immune response in certain clinical cases (Han et al., 2019) (shown in Figure 5).

### Macrophage

Macrophage is a kind of innate immune cell dwelling in various tissues with multiple subtypes, which can be classified into classically activated macrophages (M1) and alternatively activated macrophages (M2). Early researches have implied the possibility of m^6^A weakening immune response (Durbin et al., 2016). However, recent researches indicate that m^6^A modification plays an important role in antivirus, negative feedback control of macrophage activation (Du et al., 2020), and the polarization of macrophages. Roles of m^6^A Writers, Erasers, and Readers in macrophages are summarized in Table 1.

**TABLE 1:** Roles of m^6^A Writers, Erasers, and Readers in macrophages.

m^6^A regulator	Cell subtype	Target genes	Biological function
**Writers**			
METTL3	M1	*STAT1*	Promote polarization
	M2	*STAT1*	Inhibit polarization
	M1/M2	*Cgas, Ifi16, Sting, hnRNPA2B1*	Increase the production of IFN-β Amplify the immune response to DNA virus
		*Irakm*	Promote activation
METTL14	M1/M2	*Ebi3*	suppress CD8^+^ T cell dysfunction and tumor growth
**Erasers**			
FTO	M1	*STAT1*	Promote polarization
	M2	*STAT6, PPAR-γ*	
ALKBH5	M1/M2	*Mavs, Traf3, Traf6*	Inhibit translation and production of IFN
**Readers**			
YTHDF2	M1/M2	*STAT1, PPAR-γ*	Impede macrophage activation
		*MAP2K4, MAP4K4*	Inhibit the expression of pro-inflammatory cytokine and inflammatory response
YTHDF3		*FOX O 3*	Impede the expression of IFN-stimulated genes and immunity response to VSV
IGF2BP2		*TSC1, PPAR-γ*	regulate macrophage phenotypic activation and inflammatory diseases

In detail, “Writers” METTL3 catalyzes the m^6^A methylation at coding sequence (CDS) and 3′-UTR of *STAT1* mRNA, facilitating the polarization of M1, but having opposite impact on M2 (Liu Y et al., 2019). Another study has also illustrated METTL3’s function in promoting M1 differentiation, which afterwards benefits bone marrow mesenchymal stem cells (Lei et al., 2021). Moreover, METTL3 can methylate hnRNPA2B1 and *Cgas*, *Ifi16*, and *Sting* mRNA simultaneously, and then the affinity of hnRNPA2B1 to three mRNA above is improved, which ultimately increases the production of IFN-β and amplifies the immune response to DNA virus (Wang L et al., 2019). In addition, METTL3 deficiency have proved to impede the activation of macrophages through TLR4 signaling pathway by stabilizing *Irakm* transcripts (Tong et al., 2021). As for METTL14, there is also indirect evidence indicating its function in suppressing CD8^+^ T cell dysfunction and tumor growth (Dong et al., 2021).

After knockout of “Erasers” *FTO*, the polarization of both M1 and M2 can be inhibited, during which the expression of STAT1 in M1 was decreased, while the degradation of *STAT6* and *PPAR-γ* mRNA is increased (Gu et al., 2020). Furthermore, ALKBH5 demethylate DDX46-binded *Mavs*, *Traf3*, and *Traf6* mRNAs, leading to the retention of these mRNAs inside nuclear, indirectly inhibiting translation and reducing the production of IFN. In the end, antiviral activation of macrophages is weakened (Zheng et al., 2017).

Knockdown of “Readers” *YTHDF2* can stimulate the expression of *STAT1* (Huangfu et al., 2020) and *PPAR-γ* mRNA (Gu et al., 2020), while forced expression of YTHDF2 could destabilize *MAP2K4* and *MAP4K4* mRNA and activate the NF-κB and MAPK pathway (Yu et al., 2019), which will facilitate LPS-induced osteoclastogenesis and inflammatory response (Fang et al., 2021). YTHDF3 was regarded as a negative regulator of antivirus. With the assistance of PABP1 and elF4G2, YTHDF3 can bind the translation initiation site of *FOXO3* mRNA and promote translation. As a result, the expression of IFN-stimulated genes is inhibited, which weakens the immunity response to Vesicular stomatitis virus (VSV) (Zhang Y et al., 2019). Moreover, another reader IGF2BP2 has also proved to be associated with the phenotypic activation of macrophage (Wang X et al., 2021).

Researches focusing on m^6^A modification in macrophages reveal its association with some diseases. The occurrence of atherosclerosis (AS) has proved to be linked with m^6^A modification. During this progress, ox-LDL induces the expression of DDX5 in macrophages and limits the function of METTL3 which transfers the methyl group to macrophages scavenger receptor A (*MSR1*) mRNA. Ultimately, *MSR1* mRNA is stabilized, and more MSR1 is synthesized. Uptake of more lipids further facilitates the formation of foam cells, resulting in the progression of AS (Zhao W et al., 2018). In another study, Zhang X et al. (2021) has also identified the function of METTL3 in promoting ox-LDL-induced inflammation and mitochondrial dysfunction by methylating peroxisome proliferator-activated receptor-γ coactivator 1-alpha (PGC-1α) mRNA with the assistance of YTHDF2. Intriguingly, m5C, another form of RNA methylation, can deteriorate AS induced by hyper-homocysteinemia (Wang et al., 2017), while acute coronary syndrome, whose main pathological basis is AS, is also related to the m^6^A modification of circ-0029589 in macrophage (Guo M et al., 2020).

The dysfunction of m^6^A in macrophages is also a pathogenesis factor of autoimmune diseases. Wang J et al. (2019) found that in patients with rheumatoid arthritis, METTL3 in macrophages is obviously improved and positively associated with CRP and ESR. Moreover, lipopolysaccharide (LPS) can stimulate the expression of METTL3 in macrophages and then slack the immune response to inflammation through NF-κB pathway (Wang J et al., 2019). Other autoimmune diseases such as osteoarthritis (Liu Q et al., 2019) and SLE (Li et al., 2018) show possibility of having connection with m^6^A dysfunction in macrophages.

## The Application of m^6^A Modification and its Development Prospect

Given the identification of aberrant m^6^A modification in various diseases, targeting m^6^A machinery in specific cells can be regarded as a new treatment for viral infection, cancer, and autoimmune diseases. However, changes in m^6^A levels in different diseases are lack of consistency, so treatment targeting m^6^A should be supposed to modulating m^6^A level to normal level, instead of simply accelerating or decelerating m^6^A modification (Wang et al., 2018).

### m^6^A-Associated Proteins Modulating DNA Repair

Plenty of researches have confirmed the correlation between m^6^A and DNA repair in different situations. Zhang et al. have reported the METTL3-m^6^A-YTHDC1 axis which promotes the double-strand breaks by modulating DNA-RNA hybrid accumulation (Zhang et al., 2020). Other researches indicate that accumulation of the three-stranded R-loops, formed by RNA: DNA hybrid and single stranded DNA, is regulated by YTHDF2 (Abakir et al., 2020), METTL3, and tonicity-responsive enhancer binding protein (TonEBP) (Kang et al., 2021). More surprisingly, the arginine substrates of METTL14 itself at intrinsically disordered C terminus can also be methylated. It proved to be an initial signal of interaction between METTL14 and RNA polymerase II, which will afterwards implement the m^6^A modification of target mRNA. Subsequent studies have confirmed that METTL14 arginine methylation is associated with the enhanced translation of DNA repair genes (Wang Z et al., 2021). Recent studies even clarified the correlation between DNA damage repair and m^6^A-modified retrotransposable element (RTE) RNAs, in which intronic Long Interspersed Element-1 (LINE-1) interacts with the hosting gene transcription, resulting in the downregulation of its expression (Xiong et al., 2021). KIAA1429 was also recently discovered to have close links with the modulation of response to cisplatin in germ cell tumor by interfering with DNA damage response (Miranda-Gonçalves et al., 2021).

As for Erasers, the homologs of AlkB (ALKBHs), which originally function as repair proteins in *E. coli*, are also important regulators of DNA repair and m^6^A modification at the same time in mammalian cells (Müller et al., 2017; Müller et al., 2018). Recently, the ERK/JNK/ALKBH5-PTMs/m^6^A axis has been reported to participate in the regulation of ROS-induced DNA damage response, in which progress IGF2BP also plays a part in extending mRNA half time (Yu et al., 2021). METTL3 and FTO can jointly regulate the m^6^A modification in RNA at DNA damage sites induced by ultraviolet. The function of DNA polymerase κ (Pol *κ*), which is the key DNA repair enzyme, require the catalytic activity of METTL3, implying the m^6^A modification directs the recruitment of Pol κ to the DNA damage sites (Xiang et al., 2017). Furthermore, METTL14 has proved to play a tumor-suppressive role in ultraviolet-induced skin tumorigenesis (Yang et al., 2021). All the m^6^A-associated proteins which regulate DNA repair are summarized in Table 2.

**TABLE 2:** m^6^A-associated proteins in DNA repair.

m^6^A regulator	Biological function
**Writers**	
METTL3	Modulate DNA-RNA hybrid accumulation
	Regulate accumulation of the three-stranded R-loops
	Regulate the m^6^A modification in ultraviolet-induced DNA damage
	Direct the recruitment of Pol κ to the DNA damage sites
METTL14	Enhance translation of DNA repair genes
	Suppress ultraviolet-induced skin tumorigenesis
KIAA1429	Interfere with DNA damage response in cisplatin-treated germ cell tumor
**Erasers**	
FTO	Regulate the m^6^A modification in ultraviolet-induced DNA damage
ALKBH5	Regulate ROS-induced DNA damage response
**Readers**	
YTHDC1	Modulate DNA-RNA hybrid accumulation
YTHDF2	Regulate accumulation of the three-stranded R-loops
IGF2BP2	Extend mRNA half time in ROS-induced DNA damage response

In conclusion, since m^6^A, along with its associated proteins, plays an important role in the pathogenesis and facilitating DNA repair, attaching m^6^A-targeted therapy to traditional chemo- or radiotherapy may improve the prognosis of some diseases such as carcinoma through two mechanisms.

### Medication Targeting m^6^A

Abnormally elevated m^6^A level is the feature of most malignant tumor, so developing new drugs inhibiting m^6^A modification is the most fundamental idea to treat these diseases. Actually, this kind of drugs has been developed for decades. 3-Deaza-Adenosine (DAA) with its analogue can block S-adenosylhomocysteine (SAH) hydrolase, which results in the accumulation of SAH and feedback suppression of SAM (Chiang, 1998). So DAA can indirectly inhibit the m^6^A modification of mRNA. However, since DAA can suppress the m^6^A modification in many physiological or pathological processes, there will be many unexpected side effects, such as the prevention of T lymphocyte activation, hypotensive effect, and activation of gene expression. At present, DAA is mainly used for the treatment of AIDS (Kennedy et al., 2016).

Moreover, some diseases like acute myeloid leukemia (AML) are characterized by the aberrant decrease of m^6^A (Li Z et al., 2017). Rhein (Chen et al., 2012), curcumin (Lu N et al., 2018), meclofenamic acid (Huang et al., 2015), and Saikosaponin-D (Sun et al., 2021) can inhibit the function of FTO by binding the active site of FTO or m^6^A position of mRNA. Two emerging small molecules targeting FTO demethylase called FB32 and FB32-2, which can dramatically inhibit the progression of AML cells *in vitro* and *in vivo*, have been developed recently (Huang et al., 2019). Coupled with the latest advancement called STM2457, which is a highly potent and selective first-in-class catalytic inhibitor of METTL3 (Yankova et al., 2021), we can foresee that the era of treating AML or other diseases featured by m^6^A level unbalance using m^6^A-targeted method is coming.

### Immunotherapy Targeting m^6^A

Although solid researches data is unavailable, the regulatory function of m^6^A modification to immunocytes makes it possible for m^6^A to be a new target for immunotherapy. It has been demonstrated that m^6^A is a regulator of Tn differentiation, T lymphocyte homeostasis (Li H-B et al., 2017), and suppressive function of Tregs (Tong et al., 2018). Recent studies have found that loss of METTL3 in myeloid cells reprograms the macrophages and increases Treg infiltration into tumors by influencing the YTHDF1-mediated translation of SPRED2 (Yin et al., 2021). Therefore, inhibiting mRNA m^6^A modification of Tregs at tumor site or myeloid cells can motivate the antitumor activation of CD8^+^ T cell, which can be a promising immunotherapy. Meanwhile, inhibiting mRNA m^6^A modification in Tn can reduce the formation of effector T cell, which is helpful for the treatment for autoimmune diseases. Furthermore, regulating the expression of METTL3 (Wang H et al., 2019) and YTHDF1 to a suitable level can improve DCs’ ability of presenting tumor neoantigen. More importantly, depletion of YTHDF1 and blockade of checkpoint have a synergistic effect on strengthening antitumor immunity (Han et al., 2019), and ALKBH5 (Li N et al., 2020) and METTL3/14 (Wang et al., 2020) have also proved to regulate anti PD-1 response. So as for patients resistant to the PD-1 immunotherapy, targeting m^6^A can be a new alternative treatment. Since m^6^A decoration of viral double-stranded RNA can also downregulate the innate sensing pathway of antiviral response (Qiu et al., 2021), immunotherapy targeting m^6^A is bound to be a promising therapy for various diseases including viral infections, autoimmune disorders, cancers, etc.

## Discussion

As the most abundant post-transcriptional mRNA modification in mammals, m^6^A is involved in the occurrence of several diseases. Recently, an enormous amount of m^6^A related studies in immunocytes highlight the fact that targeting m^6^A can be a promising new treatment strategy for viral infection, cancer, and autoimmune diseases. However, in different cell lines, diseases, even different types of the same diseases, the changes of m^6^A level, as well as functions of three m^6^A-associated enzymes lack consistency. Moreover, m^6^A modification is widely involved in a variety of cellular processes and medication targeting m^6^A is not selective. All these reasons indicate that clinical treatment *via* targeting m^6^A modification may be not safe enough. Thus, there is unmet need to develop more sophisticated techniques for m^6^A detection (Dai et al., 2018; Castellanos-Rubio et al., 2019). Moreover, detailed studies on disease mechanisms are required to realize the clinical application of m^6^A-targeting treatment. Pharmaceutical researches on drugs with high selectivity or combination of existing drugs and targeted drug delivery system can also promote the accurate treatment of diseases through the m^6^A-targeting method. Some recent experimental results have indicated the promising prospects of this field (Zhu et al., 2021). Last but not least, m^6^A modification is supposed to be highly relevant to gut microbiota (Jabs et al., 2020), heat shock proteins (Feng et al., 2020), sepsis (Sun et al., 2020; Xing et al., 2021), and pulmonary hypertension (Pan et al., 2020) and even peripheral nerve injury (Zhang L et al., 2021). These studies can provide valuable experimental basis for development of new treatments.
